# Investigating Pregnant Women’s Attitudes toward Herbal Remedies: A Cross-Sectional Study

**DOI:** 10.3390/ijerph21101290

**Published:** 2024-09-27

**Authors:** Deniz Al-Tawalbeh, Falastine Hamdan, Eshraq Al-Momani, Huda Atiyeh, Amal Mayyas

**Affiliations:** 1 Department of Medicinal Chemistry and Pharmacognosy, Faculty of Pharmacy, Yarmouk University, Irbid 21163, Jordan; 2 Faculty of Nursing, Al-Balqa Applied University, Al-Salt 19117, Jordan; falsteen.hamdan@bau.edu.jo; 3 Department of Applied Science, Irbid University College, Al-Balqa Applied University, Irbid 1293, Jordan; esshraq.momani@bau.edu.jo; 4 Department of Adult Health, Nursing College, Zarqa University, Zarqa 2000, Jordan; hatiyeh@zu.edy.jo; 5 Faculty of Health Science, American University of Madaba, Madaba 11821, Jordan; a.mayyas@aum.edu.jo

**Keywords:** pregnancy, herbal remedies, ethnopharmacology, safety, infants

## Abstract

Background: Herbal remedies play a significant role in daily life, regardless of region or ethnicity. While they offer various health benefits, they may also pose risks, including toxicity and undesirable side effects. Pregnant women, one of the most vulnerable populations, frequently use herbal remedies, often without informing their healthcare providers, which can lead to unforeseen consequences for both the mother and the fetus. Method: A total of 590 women participated in an online survey designed to assess demographic factors, awareness of herbal remedies during pregnancy, and the potential impact of these remedies on maternal and fetal health. Results: The survey revealed that 35.8% of the participants used herbal remedies during pregnancy. The most common reasons for their use were beliefs in their safety and family recommendations. Anise (*Pimpinella anisum* L.) was the most frequently used herb. Conclusions: The findings indicate that pregnant women generally have limited knowledge about herbal remedies and their potential risks. To mitigate this, it is essential to develop and disseminate comprehensive safety and efficacy guidelines. Both physicians and pregnant women should be well-informed to ensure the protection of maternal and fetal health.

## 1. Introduction

Pregnancy is a unique period marked by physiological changes that impact every system in the body [1]. These changes, largely driven by hormonal shifts in estrogen, progesterone, and the growing uterus, are considered normal. However, while many of these changes resolve themselves postpartum, they can cause discomfort and various health issues during pregnancy [2].

Mokaberinejad et al. found that 85% of pregnant women experience gastrointestinal disorders, including nausea, vomiting, constipation, and heartburn. Additionally, sleep disorders, particularly during the third trimester, are common, with reports of insomnia (20.23%), frequent waking (62.88%), and daytime drowsiness (86.35%) [3]. 

In Jordan, low income and maternal concerns about the potential side effects of medications on the fetus are significant factors contributing to the high reliance on complementary and alternative medicine (CAM) among pregnant women [4]. The National Center for Complementary and Alternative Medicine defines CAM as “a group of diverse medical and healthcare systems, practices, and products that are not currently considered part of conventional medicine” [5]. Traditional herbal medicine, a key component of CAM, is defined by the World Health Organization (WHO) as the use of naturally occurring plant resources that have not undergone industrial modification [6,7].

According to the WHO, approximately two-thirds of countries—both developed and developing—depend on herbal medicine [8]. Factors such as the availability of herbal remedies, their perceived safety, and dissatisfaction with conventional therapies contribute to the widespread use of herbal medicine [9]. Another WHO study revealed that 80% of people in rural areas in developing countries rely on traditional remedies during pregnancy to meet their health needs [10].

Herbal remedies are frequently used by expectant mothers to ease the physiological changes associated with pregnancy [10]. Research on pregnant and breastfeeding women indicates that herbs are commonly used to address conditions, such as nausea, vomiting, anxiety, and anemia, and to boost milk production [11]. Studies conducted in the United States report that the prevalence of herbal use during pregnancy is 20–60% [12]. This widespread use is likely driven by the belief that herbs are safe, enhance well-being, and align with cultural and religious practices [13]. 

In Jordan, as in several other countries, women utilize herbs for a range of purposes, from culinary uses as spices to medicinal uses as herbal remedies [14]. Jordan’s diverse climate and geographical features support the growth of a wide variety of herbal plants. The country is home to more than 2000 plant species across around 700 genera, with approximately 485 species from nearly 99 plant families classified as medicinal ones [15,16]. Numerous studies in Jordan have investigated the prevalence of herbal remedy use, revealing that cultural beliefs, ease of access, and the absence of health insurance drive individuals to prefer herbal remedies over conventional medical treatments [17].

Ethnopharmacological studies from different regions of Jordan have highlighted the use of *Peganum harmala* L. and *Majorana hortensis* L. to facilitate labor [18,19], *Verbena officinalis* L. to enhance milk secretion [20], *Ferula asafoetida* to induce abortion, and *Lavandula officinalis* to prevent miscarriage [19].

However, there is limited research on the use of herbal remedies among pregnant women in Jordan, and data on their safety and efficacy remains sparse. Given this lack of information, the aim of this study is to examine Jordanian women’s attitudes toward the use of herbal remedies during pregnancy and identify the factors that influence their preference for herbal treatments over conventional medicine. Additionally, this study seeks to identify the most commonly used herbs, assess their pharmacological effects as reported in the literature, and determine the most frequently treated conditions with herbal remedies during pregnancy.

## 2. Materials and Methods

### 2.1. Study Design

A cross-sectional, self-reported online questionnaire survey was conducted, targeting pregnant women residing in Jordan. The questionnaire, developed using Google Forms, was designed to facilitate data collection on the most commonly used herbal remedies by Jordanian women and the factors influencing their use. Invitations were extended to women who were currently pregnant or had been pregnant previously using a social media platform and a snowball sampling method. The questionnaire link was included with the invitation, and participants were informed beforehand that the survey was anonymous, voluntary, and that their data would remain confidential. The study’s purpose was briefly explained, and the questionnaire was initially created in English, translated into Arabic, and then back-translated by a bilingual speaker to ensure translation accuracy. 

### 2.2. Questionnaire Development

A literature review provided the basis for developing a set of key topics and items relevant to the study. These topics were reviewed by experts in pharmacognosy and nurses specializing in pregnancy to ensure content validity. Following this review, a pilot study was conducted with 50 Jordanian women to assess the reliability of the questionnaire; these responses were not included in the final study sample. The final survey featured closed-ended questions with multiple-choice responses and was divided into four sections: demographics, pregnancy-related information, herbal preparation use, and perceived risks. Demographic information comprised age, occupation, education, place of residence, total income, and health insurance. Pregnancy-related information was gathered through questions about the number of births, current pregnancy status, and experience of any minor disorders during pregnancy. The use of herbal preparations and perceived risks were assessed with questions regarding the use of herbal preparations to relieve or improve minor disorders, along with the sources of information on the use of these herbal preparations.

### 2.3. Statistical Analysis

Data were analyzed using SPSS (IBM version 25). Descriptive statistics, including frequencies and percentages for categorical variables, as well as means ± standard deviations or medians (lower–upper quartiles) for continuous variables, were used to analyze the responses. 

The association between sociodemographic data and knowledge, attitudes, and practices regarding herbal medicine was evaluated using the chi-square test. A *p*-value of less than 0.05 was considered statistically significant.

### 2.4. Ethical Approval

Ethical approval for this study was obtained from the Institutional Review Board of Yarmouk University (YU; REF: IRB/2024/232).

## 3. Results

Of the 590 women who participated in the survey, the majority (32.5%) were between 31 and 40 years of age, with 47.5% having attained a high level of education, primarily holding a bachelor’s degree. More than half of the participants (59.3%) lived in urban areas, and the vast majority (84.9%) had medical insurance. Approximately 50.8% of respondents reported a family monthly income of less than 500 JD, and 51% were unemployed. During pregnancy, 211 women reported using herbal remedies, with most utilizing more than one type of herb (Table 1).

Among women who preferred herbal remedies, 45.6% believed they were safer than conventional medications, with 59.2% choosing herbs based mainly on family recommendations. Furthermore, 56.1% of these women reported no side effects from using herbs during pregnancy.

**Figure 1:**
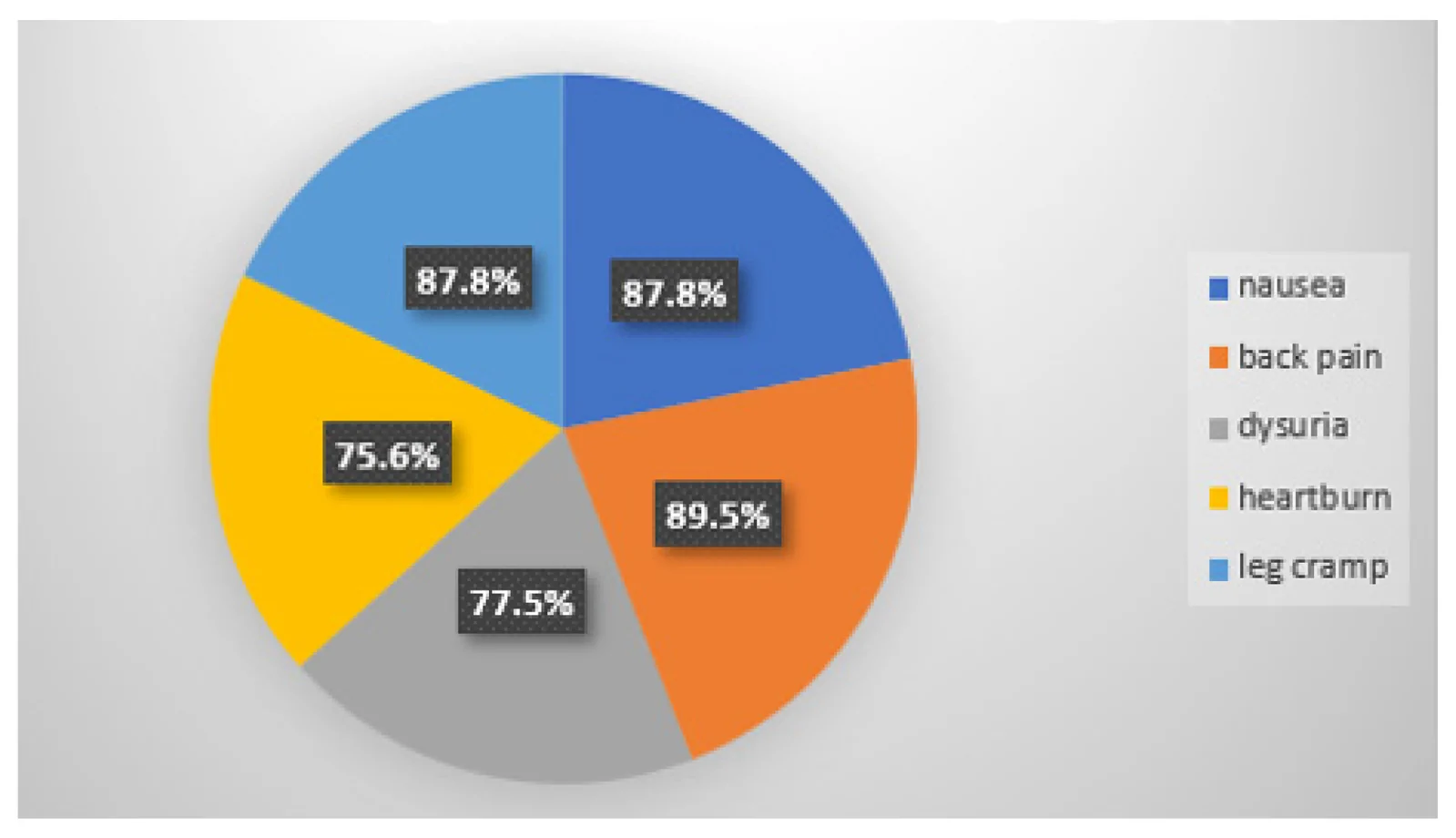
Most common health issues treated with herbs by pregnant women.

The most frequently used herb was anise (*Pimpinella anisum* L.) (47.2%), followed by peppermint (*Mentha piperita* L.) (35.3%), chamomile (*Matricaria chamomilla* L.) (34.1%), and sage (*Salvia officinalis* L.) (32.6%) (Table 2). Herbs were utilized as needed to manage pregnancy-related issues such as nausea, back pain, dysuria, heartburn, and leg cramps (Figure 1).

**Figure 2:**
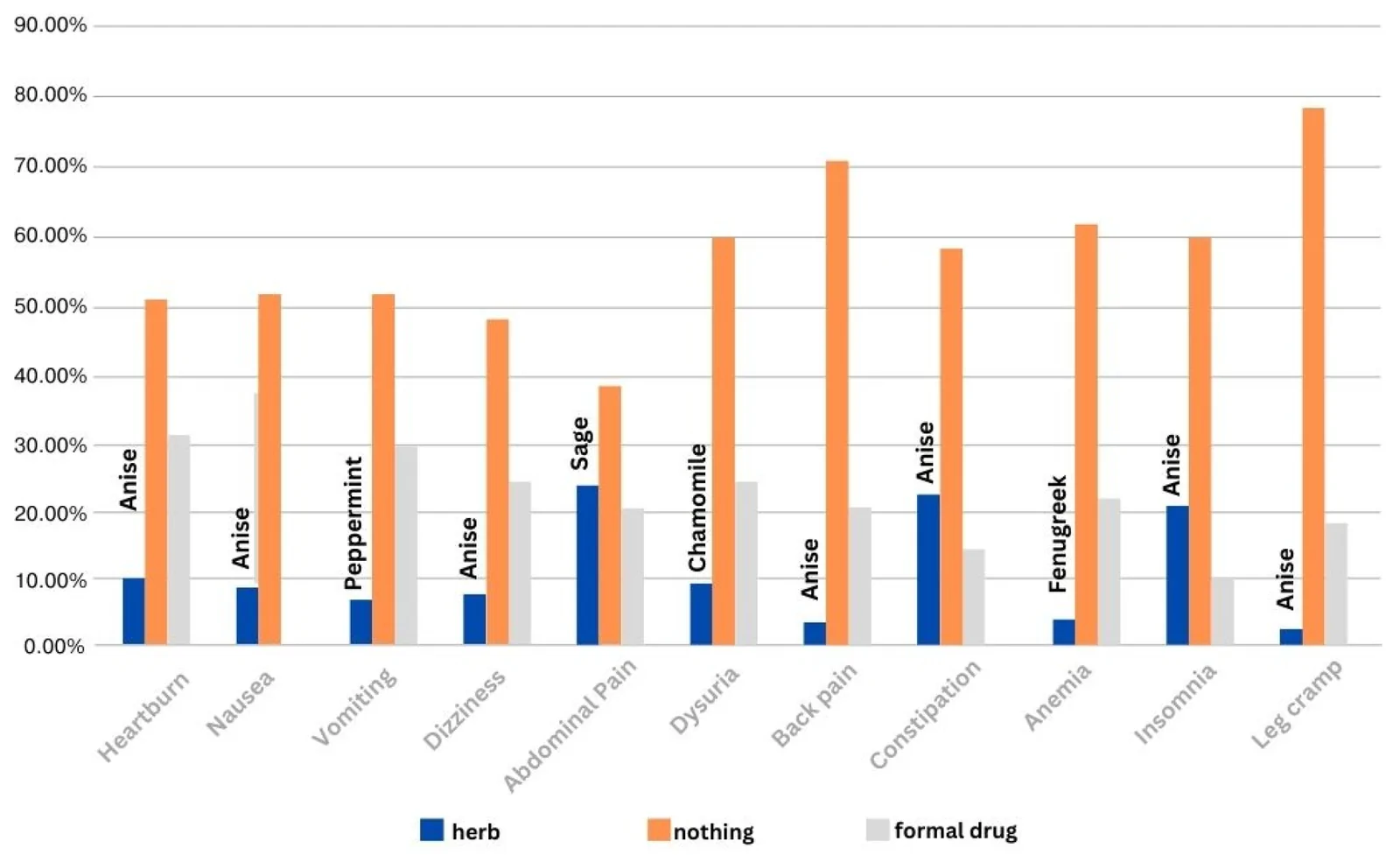
Comparison of herb use, formal medication use, and no treatment for addressing pregnancy-related issues.

As shown in Figure 2, the percentage of pregnant women who did not use any herbal or conventional medications was higher across all listed problems.

## 4. Discussion

Herbs can be beneficial for treating certain health conditions; however, they may also have harmful side effects. Therefore, it is crucial to study the herbs used by women during pregnancy and assess their safety and efficacy for both the mother and the fetus. In the present study, 35.8% of participants reported using at least one herbal remedy during pregnancy, which is lower than the 73.8% reported in a previous study conducted in Jordan in 2010 [21]. Additionally, the usage rates in neighboring countries such as Palestine and Egypt were higher at 40.0% and 45.8%, respectively [22,23]. Several factors may explain this discrepancy: the high level of knowledge among participants about herbs and their potential side effects, the relatively high educational attainment (52.1% with a bachelor’s degree), previous experiences with herbal remedies and their side effects, and the ease of accessing information online (37.8%). These results contrast with previous studies that found a higher education level and greater herbal knowledge were associated with increased use of alternative therapies among pregnant women [24]. 

In our study, 31.8% of pregnant women reported experiencing side effects from herbal medicine use, whereas 50.7% reported no side effects. The lack of reported adverse effects may explain why pregnant women prefer herbal products over conventional medications. They perceive herbal remedies as safer (45.6%), more accessible (44.9%), and more affordable (18.5%) than formal medications. This finding aligns with a Palestinian study where 82.5% of pregnant women considered herbal medicine safer than other medications [22] and an Australian study where pregnant women preferred herbal remedies for their perceived safety [25].

As stated by Tapatapaee et al. (2011), approximately 82.3% of pregnant women used herbal medicine, with 87.3% obtaining their knowledge from family members [26]. Another study found that 61.6% of women received information from their mother-in-law [21]. These findings align with our results, where 59.2% of pregnant women reported obtaining their knowledge of herbal remedies from family members, followed by online searches (37.8%).

In our survey, the most commonly used herbs were anise (47.2%), mint (35.3%), chamomile (34.1%), and sage (32.6%), while the use of conventional medications was reported at 25.8%. This finding contrasts with that of Al-Ramahi et al.’s study (2013), where chamomile (53.3%) was the most commonly used herb, followed by sage (45.8%) [22]. Another study in Palestine identified peppermint, sage, and anise as among the most frequently used herbs [27]. Although anise is the most popular herb in our survey, it may interact with warfarin, potentially enhancing its effects; thus, pregnant women should be cautious about the safety of anise during pregnancy [28]. Conversely, an Italian study associated chamomile use with an increased risk of miscarriage and preterm labor [29].

Participants reported that the high use of sage, anise, and fenugreek was due to their availability at home (55.5%). Literature recommends using sage only after 37 weeks of pregnancy, as it may induce labor and stimulate oxytocin release [30]. However, Mannion and Mansell (2012) have contraindicated fenugreek during pregnancy [31]; this is due to its hypoglycemic effect and its potential to induce oxytocin secretion [32]. 

Among the top five herbs reported in our survey, cinnamon was used for relieving abdominal pain and promoting relaxation. Other studies have highlighted cinnamon’s utility in treating gastrointestinal issues such as bloating [33] and in controlling blood sugar levels [34].

Our survey found that anemia was not commonly treated with herbal remedies, which may be attributed to the routine prescription of folic acid and multivitamins by physicians from the early stages of pregnancy [35,36].

## 5. Limitations

In this study, our goal was to reach approximately 1000 participants; however, we were able to include only 590. This shortfall may be attributed to the online nature of the survey, which may not fully represent the target population. Several limitations should be acknowledged. First, an internet-based study can introduce biases related to the demographic characteristics of the participants. Second, the survey did not include questions about the duration of herbal remedy use or the specific trimester in which these remedies were used. Last is the lack of inquiry into whether pregnant women consulted their physicians about herbal remedies. It is possible that women might not disclose their use of herbal remedies to healthcare providers, fearing that physicians might advise them to switch to conventional medications. These limitations should be considered when interpreting the results of this study. 

## 6. Conclusions

In conclusion, the widespread use of herbal remedies during pregnancy, combined with a lack of comprehensive information regarding their safety and potential risks to both mother and fetus, presents a significant public health concern. It is crucial to prioritize research and documentation on the safety and efficacy of these remedies, as well as to evaluate their potential side effects on pregnant women and their developing babies. Despite the participants’ high education levels and access to medical insurance, the strong cultural influence driving the use of herbal remedies highlights the need for greater awareness and education.

In Jordan, as in several other countries, herbal remedies are easily accessible and commonly used in households. This calls for healthcare providers to stay informed about the benefits, risks, and potential drug–herb interactions to provide appropriate guidance to pregnant women. Additionally, regulatory measures should be strengthened to limit the sale of potentially harmful herbs to pregnant women. Public health initiatives, including awareness campaigns and educational programs, are essential to inform women about the safe use of herbal remedies during pregnancy and to mitigate potential risks.

## Figures and Tables

**Table 1:** Association between sociodemographic characteristics and herbal remedy use during pregnancy.

Variable	User (*N* = 211) (*n*, (%))	Non-User (*N* = 370) (*n*, (%))	*p*-Value
Age (years)			0.609
Less than 20	3 (1.4)	7 (1.8)	
20–30	63 (29.9)	125 (33.0)	
31–40	77 (36.5)	115 (30.3)	
41–50	48 (22.7)	89 (25.9)	
More than 50	20 (9.5)	34 (9.0)	
Educational level			0.333
Basic	3 (1.4)	9 (2.4)	
Secondary	35 (16.6)	88 (32.2)	
Diploma	38 (18.0)	66 (17.4)	
Bachelor	110 (52.1)	170 (44.9)	
Master	20 (9.5)	33 (8.7)	
PhD	5 (2.4)	13 (3.4)	
Current job			0.115
Student	16 (7.6)	17 (4.5)	
Employee	90 (42.7)	124 (37.5)	
Retired	10 (4.7)	14 (3.7)	
Unemployed	95 (45.0)	206 (54.4)	
Living place			0.764
City	121 (57.3)	229 (60.4)	
Village	86 (40.8)	143 (37.7)	
Badia	4 (1.9)	7 (1.8)	
Medical insurance			0.399
Yes	179 (84.8)	322 (85.0)	
No	32 (15.2)	57 (15.0)	
Family monthly income			0.893
≤500 JD	106 (50.2)	184 (48.5)	
˃500 JD	105 (49.8)	195 (51.5)	

Significant level at α ≤ 0.05.

**Table 2:** Most frequently used herbs and their reported reason for use (*N* = 211).

Scientific Name	Common Name	Percentage (%)	Reason for Use
*Pimpinella anisum* L. *Apiaceae*	Anise	47.2	Constipation, insomnia, nausea, leg cramps, heartburn, lower back pain
*Mentha piperita* L. *Lamiaceae*	Peppermint	35.3	Heartburn, abdominal pain, nausea, vomiting, insomnia
*Matricaria chamomilla* L. *Asteraceae*	Chamomile	34.1	Abdominal pain, dizziness, heartburn, insomnia, constipation
*Salvia officinalis* L. *Lamiaceae*	Sage	32.6	Abdominal pain, nausea, vomiting, constipation, heartburn
*Cinnamomum verum J. Presl. Lauraceae*	Cinnamon	13.9	Abdominal pain, anemia, dizziness

## Data Availability

Data are available upon request from the author.
